# Epidemiological and Genetic Characteristics of Porcine Reproductive and Respiratory Syndrome Virus in South China Between 2017 and 2021

**DOI:** 10.3389/fvets.2022.853044

**Published:** 2022-04-08

**Authors:** Kui Fang, Shudan Liu, Xiangmin Li, Huanchun Chen, Ping Qian

**Affiliations:** ^1^State Key Laboratory of Agricultural Microbiology, College of Veterinary Medicine, Huazhong Agricultural University, Wuhan, China; ^2^The Cooperative Innovation Center for Sustainable Pig Production, Wuhan, China; ^3^Key Laboratory of Development of Veterinary Diagnostic Products, Ministry of Agriculture of the People's Republic of China, Wuhan, China

**Keywords:** porcine reproductive and respiratory syndrome virus, epidemiological characteristics, genetic characteristics, MLV-derived isolates, recombination

## Abstract

Porcine reproductive and respiratory syndrome virus (PRRSV) remains a major threat to the swine industry in China and has caused enormous losses every year. To monitor the epidemiological and genetic characteristics of PRRSV in South China, 6,795 clinical samples from diseased pigs were collected between 2017 and 2021, and 1,279 (18.82%) of them were positive for PRRSV by RT-PCR detecting the ORF5 gene. Phylogenetic analysis based on 479 ORF5 sequences revealed that a large proportion of them were highly-pathogenic PRRSVs (409, 85.39%) and PRRSV NADC30-like strains (66, 13.78%). Furthermore, 93.15% of these highly-pathogenic strains were found to be MLV-derived. We next recovered 11 PRRSV isolates from the positive samples and generated the whole genome sequences of them. Bioinformatic analysis showed that seven isolates were MLV-derived. Besides, six isolates were found to be recombinant strains. These eleven isolates contained different types of amino acid mutations in their GP5 and Nsp2 proteins compared to those of the PRRSVs with genome sequences publicly available in GenBank. Taken together, our findings contribute to understanding the prevalent status of PRRSV in South China and provide useful information for PRRS control especially the use of PRRSV MLV vaccines.

## Introduction

Porcine reproductive and respiratory syndrome virus (PRRSV), the etiological agent of porcine reproductive and respiratory syndrome (PRRS), is a small, enveloped, single-stranded positive-sense RNA virus belonging to the order *Nidoviridales*, family *Arteriviridae* (1, 2). PRRSV possesses a genome ~15 kb in length and contains at least ten open reading frames (ORFs), including ORF1a, ORF1b, ORF2a, ORF2b, ORF3, ORF4, ORF5, ORF5a, ORF6, and ORF7. Among these ORFs, the ORF1a and ORF1b encode two poly-proteins which are further cleaved into 16 nonstructural proteins (Nsp), including Nsp1α, Nsp1β, Nsp2, Nsp2TF, Nsp2N, Nsp3-6, Nsp7α, Nsp7β, and Nsp8-12. The remaining eight ORFs encode the viral structural proteins GP2a, E (2b), GP3, GP4, GP5, GP5a, M, and N, respectively (3–6). Within the whole genome, Nsp2 and ORF5 genes are the most variable regions, and they are commonly considered as important targets for analyzing the genetic variation and molecular epidemiology of PRRSV (5, 7, 8).

PRRSV is mainly divided into two genotypes, PRRSV2 represented by VR2332 (Accession no. AY150564), and PRRSV1 represented by Lelystad virus (Accession no. M96262) (9). The two types of PRRSV only exhibit about 50–60% nucleotide sequence identity (9–11). PRRSV2 strains are predominant in Chinese swine herds since their initial emergence in 1996 (12–14), which are further divided into nine lineages based on the ORF5 sequence (8, 15, 16). The strains circulating in China mainly cluster into four lineages: lineage 8 strains which are also called highly-pathogenic PRRSV strains represented by TJ (Accession no. EU860248), JXA1 (Accession no. EF112445), HuN (Accession no. JF268674), and CH-1a (Accession no. AY032626), lineage 5 strains which are also called classic PRRSV strains represented by VR2332, lineage 3 strains which are also called QYYZ-like strains represented by QYYZ (Accession no. JQ308798) and GM2 (Accession no. JN662424), and lineage 1 strains which are also called NADC30-like strains represented by NADC30 (Accession no. JN654459) and NADC34 (Accession no. MF326985) (15, 17). Lineage 8 is further divided into lineage 8.1 (Chinese classic PRRSV strains, represented by CH-1a) and lineage 8.5 (Chinese highly-pathogenic PRRSV strains, represented by TJ, JXA1, and HuN). Lineage 8.1 strains first emerged in China in 1996 (low virulence), while lineage 8.5 strains first emerged in China in 2006 causing severe disease characterized by high fever (40–42°C), high morbidity, high mortality in all ages of pigs, and abortion storm in sows (17–19). Lineage 3 strains first emerged in China in 2010 and mainly circulated in South China (20, 21). Lineage 1 strains first emerged in China in 2013 originated from the American epidemic strain NADC30 (22–24).

PRRSV is one of the most rapidly evolving RNA viruses through the accumulation of mutations and recombinations among different members of lineages, which have further increased the genetic diversity and complexity of PRRSVs in China (17, 25). For example, RespPRRS MLV vaccine strain (Accession no. AF066183) recombined with QYYZ led to a novel strain GM2 in South China (20, 26), and NADC30-like strains were recombinant viruses between NADC30 and Chinese highly-pathogenic strains (27). Therefore, it is important to monitor the genetic characteristics of PRRSV strains currently circulating. In this study, we investigated the epidemiological characteristics of PRRSV in South China between 2017 and 2021. In addition, eleven PRRSV strains were successfully isolated and the complete genomes were sequenced and analyzed.

## Materials and Methods

### Sample Collection and Pre-treatment

Between the year 2017 and 2021, a total of 6,795 clinical samples including lung tissues, lymph nodes, stillbirths, and sera were collected from diseased pigs suspected of PRRSV infection in Guangxi and Guangdong province. The clinical symptoms include abortion of pregnant sows, respiratory disease of Post-weaned or growing pigs. The tissue samples were homogenized in Dulbecco's modified Eagle's medium (DMEM, Gibco) using a TissueLyser II (QIAGEN) and diluted in phosphate-buffered saline (PBS) at a ratio of 1:10, then frozen at −80°C and thawed three times and centrifuged at 5,000 rpm for 5 min. The supernatants and sera were stored at −80°C for RNA extraction and virus isolation.

### RT-PCR Detection and Sequencing

Total viral RNA was extracted using TRIzol reagent (Invitrogen, USA) according to the manufacturer's instructions and dissolved in RNase-free water. cDNA was synthesized using a M-MLV Kit with random hexanucleotide primers (TaKaRa Co. Dalian, China) following the manufacturer's instructions. A pair of specific primers targeting the ORF5 gene was synthesized based on a previous study (12) (Table 1). Thermocycler conditions used for PCR were 5 min at 95°C followed by 35 cycles of 30 s at 95°C, 30 s at 60°C and 1 min at 72°C, and a final extension of 10 min at 72°C. The products were visualized by electrophoresis on a 1.0% agarose gel containing ethidium bromide. PCR products were purified and cloned into the pMD18-T vector (TAKARA, Japan). Recombinant clones were further sequenced by Tsingke Biotech Co., LTD (Wuhan, China).

**Table 1:** Primers used in this study.

**Primer**	**Sequence (5^**′**^- 3^**′**^)**	**Size (bp)**
ORF5-F	GGCGACCGTTTTAGCCTGTCTT	735
ORF5-R	ATCATTATTGGCGTG TAGGTG	
1F	ATGACGTATAGGTGTTGGCTCTATG	1,115
1R	TCCGCTGTAGGTACTTGCCA	
2F	CCARYAYGGCTGCCTCCC	1,271
2R	AACCCCAGGAAGCACAACA	
3F	TGCTGCTGCYMTCAGAATAAA	1,537
3R	AAGCTGCAAAACCCCAATC	
4F	TCCCATCTCCCTRTTTTCTTCT	1,213
4R	GGGAGATGCACAAYCTAGACGT	
5F	CAGGRGGAGGCCCACACCT	1,437
5R	CCAGSACAGCTGGGAGAATCT	
6F	GCGAGGTRCCTTCAGATCTTTG	1,399
6R	AGCTTKGCRGCTTCCCACTG	
7F	AAGTACCAGAARTTTTGGGACAAG	1,323
7R	AGTTYTCTCGCACAGCCTGTG	
8F	CCACCCAAGGSTTTGTTTTG	1,296
8R	AGATTATRACWGGACAATGCTGGT	
9F	TCCATGTGGGAAAAACTCAGG	1,333
9R	GGAGAGAATCTACAACGCGCTT	
10F	GTGTATGACCCACAYAGGCAAYT	1,340
10R	ARGCCTAAAGTTGGTTCAATGAC	
11F	CACACCTGGGGRTTTGAATC	1,376
11R	AAGAAAGGACGCAGCCATTC	
12F	GAGATATTYGGGATAGGRAATGTG	1,270
12R	CGGAACCATCAAGCACAACT	
13F	TGCATGTCCTGGCGCTACTC	1,231
13R	AATTWCGGCCGCATGGTTC	

### Virus Isolation and Genome Sequencing

The ORF5-positive samples were used for virus isolation. The stored supernatants and sera samples were filtered through a 0.45 μm pore size membrane and transferred to porcine alveolar macrophage (PAM) cells. The cells were then incubated at 37°C for 3–5 days and monitored daily for cytopathic effects (CPE). The cultures were harvested when CPE appeared and frozen at −80°C. PAMs were obtained from 4-week-old specific-pathogen-free (SPF) pigs and cultured in RPMI 1,640 medium (Gibco BRL Co., USA) supplemented with 10% fetal bovine serum (FBS).

Genomic sequencing was performed on the third passage of the virus on PAMs. Total RNA extraction and reverse transcription were performed as described above. cDNAs were used as templates to amplify the complete genomes. Thirteen pairs of primers were designed and synthesized (Table 1). Thermocycler conditions used for PCR were 2 min at 98°C, followed by 35 cycles of 10 s at 98°C, 20 s at 60°C and 40 s at 72°C, and a final extension of 5 min at 72°C. The PCR products were purified and sequenced as described above. The obtained sequences were assembled by Seqman program of DNASTAR 7.1 software (DNASTAR Inc., Madison, WI, USA). The complete genome sequences were submitted to GenBank, and accession numbers were listed in Table 2.

**Table 2:** Information of 11 PRRSV isolates from South China in this study.

**Strain**	**Area**	**Time**	**Accession no**.
GD7666	Guangdong, China	2020	OM202893
GX505	Guangxi, China	2020	OM202894
GX1858	Guangxi, China	2020	OM202895
GX3251	Guangxi, China	2021	OM202896
GX4852	Guangxi, China	2021	OM202897
GX4934	Guangxi, China	2021	OM202898
GX5416	Guangxi, China	2021	OM202899
GX7111	Guangxi, China	2020	OM202900
GX7668	Guangxi, China	2020	OM202901
GX11045	Guangxi, China	2020	OM202902
GX11373	Guangxi, China	2020	OM202903

### Bioinformatical Analysis

Nucleotide and deduced amino acid (aa) sequences of complete genome, Nsp2, and ORF5 were aligned using MegAlign program of DNASTAR 7.1 software (DNASTAR Inc., Madison, WI, USA) to determine sequence homology. Phylogenetic analysis was conducted in MEGA 7 using the neighbor-joining method with 1,000 bootstrap replicates based on the comparative results exported from ClustalW. Reference PRRSV sequences downloaded from GenBank were listed in Table 3.

**Table 3:** Information of the PRRSV reference strains used in this study.

**Strain**	**Area**	**Time**	**Accession no**.
Lelystad Virus	Netherlands	1993	M96262
VR-2332	America	2003	AY150564
RespPRRS MLV	America	2005	AF066183
CH-1a	Beijing, China	1996	AY032626
CH-1R	Heilongjiang, China	2008	EU807840
HuN	Hunan, China	2007	JF268674
JXA1	Jiangxi, China	2006	EF112445
TJ	Tianjin, China	2006	EU860248
TJbd14-1	Tianjin, China	2014	KP742986
XJ17-5	Xinjiang, China	2017	MK759853
NADC30	America	2008	JN654459
JL580	Jilin, China	2013	KR706343
SDQZ-1609	Shandong, China	2016	MH651746
LNCH-1604	Liaoning, China	2016	MH651741
NADC34	America	2014	MF326985
CH2018NCV-Anheal-1	China	2018	MH370474
GM2	Guangdong, China	2011	JN662424
QYYZ	Guangdong, China	2011	JQ308798

The complete genome sequences were aligned using ClustalW program of MEGA 7 software for recombination screening. RDP v4.8 containing seven algorithms (RDP, BootScan, GENECONV, MaxChi, Chimera, SiScan, and 3Seq) was used to predict the putative recombination events and precise recombination breakpoints. Recombination events were only considered significant (*p*-value < ) 1 x 10^−6^) when supported by at least five of the seven detection methods (17, 28). SIMPLOT v3.5.1 with a window size of 200 bp and a step size of 20 bp was used to further check the recombination signals.

## Results

### RT-PCR Detection of PRRSV in South China

**Figure 1:**
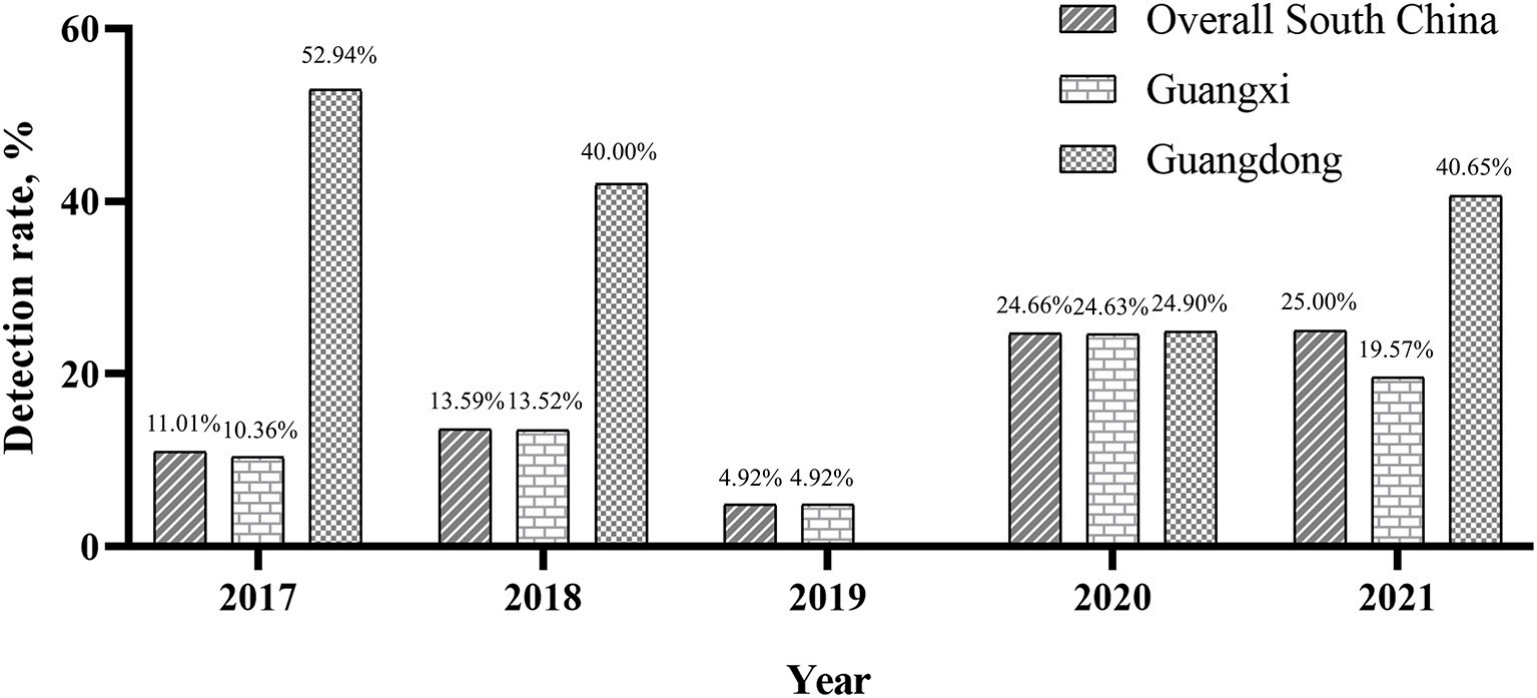
Detection rate of PRRSVs in different regions between 2017 and 2021.

Of the 6,795 clinical samples collected between the year 2017 and 2021, 1,279 (18.82%) were positive for PRRSV based on ORF5 gene detection by RT-PCR. Annually, the overall PRRSV detection rates were 11.01% (122/1,108) in 2017, 13.59% (235/1,729) in 2018, 4.92% (15/305) in 2019, 24.66% (456/1,849) in 2020, and 25.00% (451/1,804) in 2021. These results demonstrated an increasing yearly trend (Figure 1). The overall detection rates between 2017 and 2021 in Guangxi and Guangdong were 16.78% (1,017/6,059) and 35.60% (262/736), respectively. In Guangxi, the annual detection rates were 10.36% (113/1,091), 13.52% (233/1,724), 4.92% (15/305), 24.63% (394/1,600), and 19.57% (262/1,339), respectively (Figure 1). In Guangdong, the annual detection rates were 52.94% (9/17), 40.00% (2/5), 0% (0/0), 24.90% (62/249), and 40.65% (189/465), respectively (Figure 1). Monthly, the highest detection rates were observed in June (23.38%, 90/385), February (22.50%, 171/760), and January (22.14%, 122/551) (Table 4).

**Table 4:** Monthly detection rate of PRRSV in South China between 2017 and 2021.

**Month**	**Sample size**	**Positive samples size**	**Detection rate**
Jan	551	122	22.14%
Feb	760	171	22.50%
Mar	813	168	20.66%
Apr	502	89	17.73%
May	736	137	18.61%
Jun	385	90	23.38%
Jul	479	100	20.88%
Aug	498	98	19.68%
Sept	598	103	17.22%
Oct	412	67	16.26%
Nov	553	77	13.92%
Dec	508	57	11.22%

**Figure 2:**
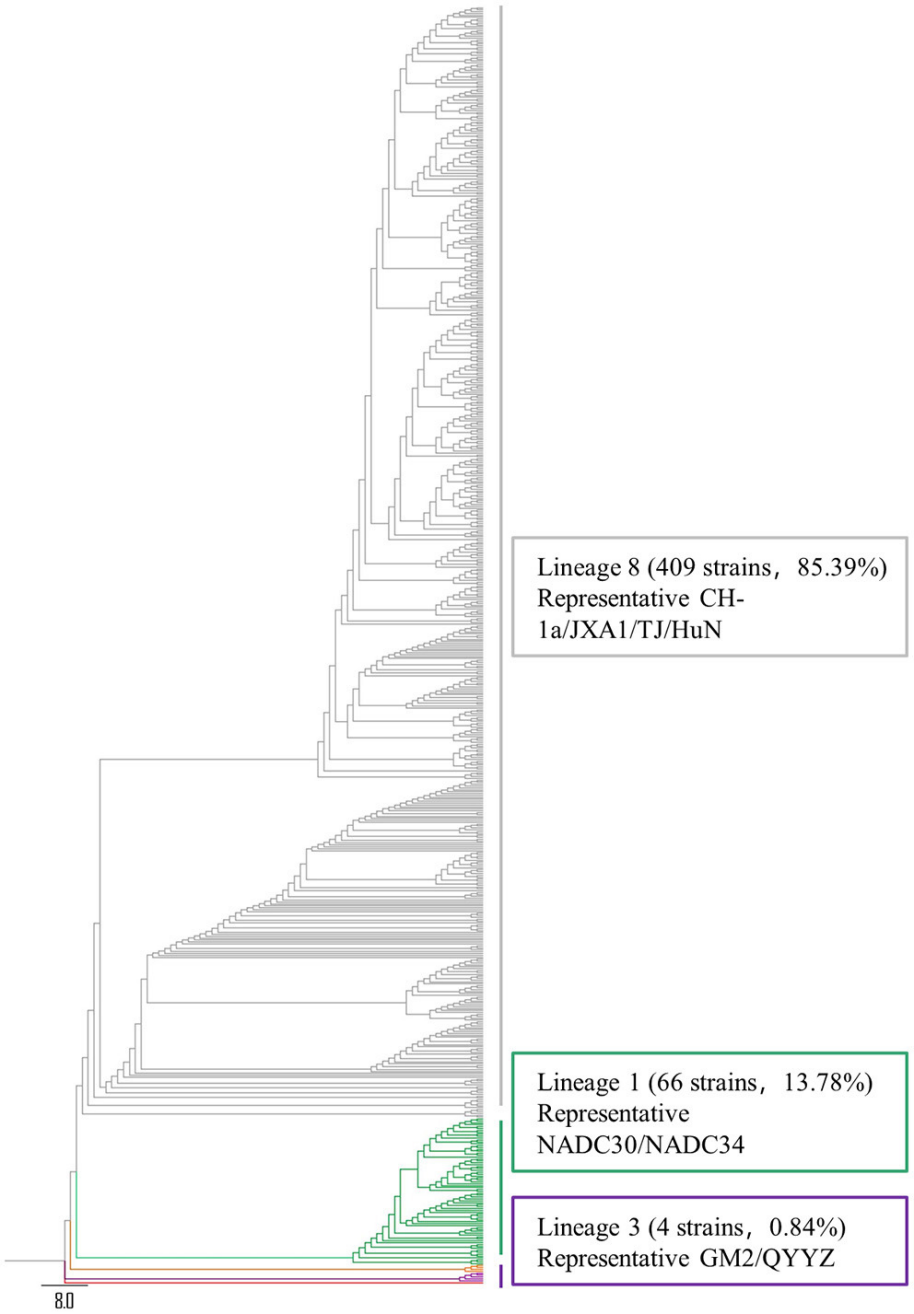
Phylogenetic tree based on ORF5 sequence. Different phylogenetic cluster are shown in different colors. Sequences were aligned using the MegAlign program of DNASTAR, followed by phylogenetic analysis in MEGA 7 by using Neighbor-Joining method with bootstrap 100 times (29).

**Figure 3:**
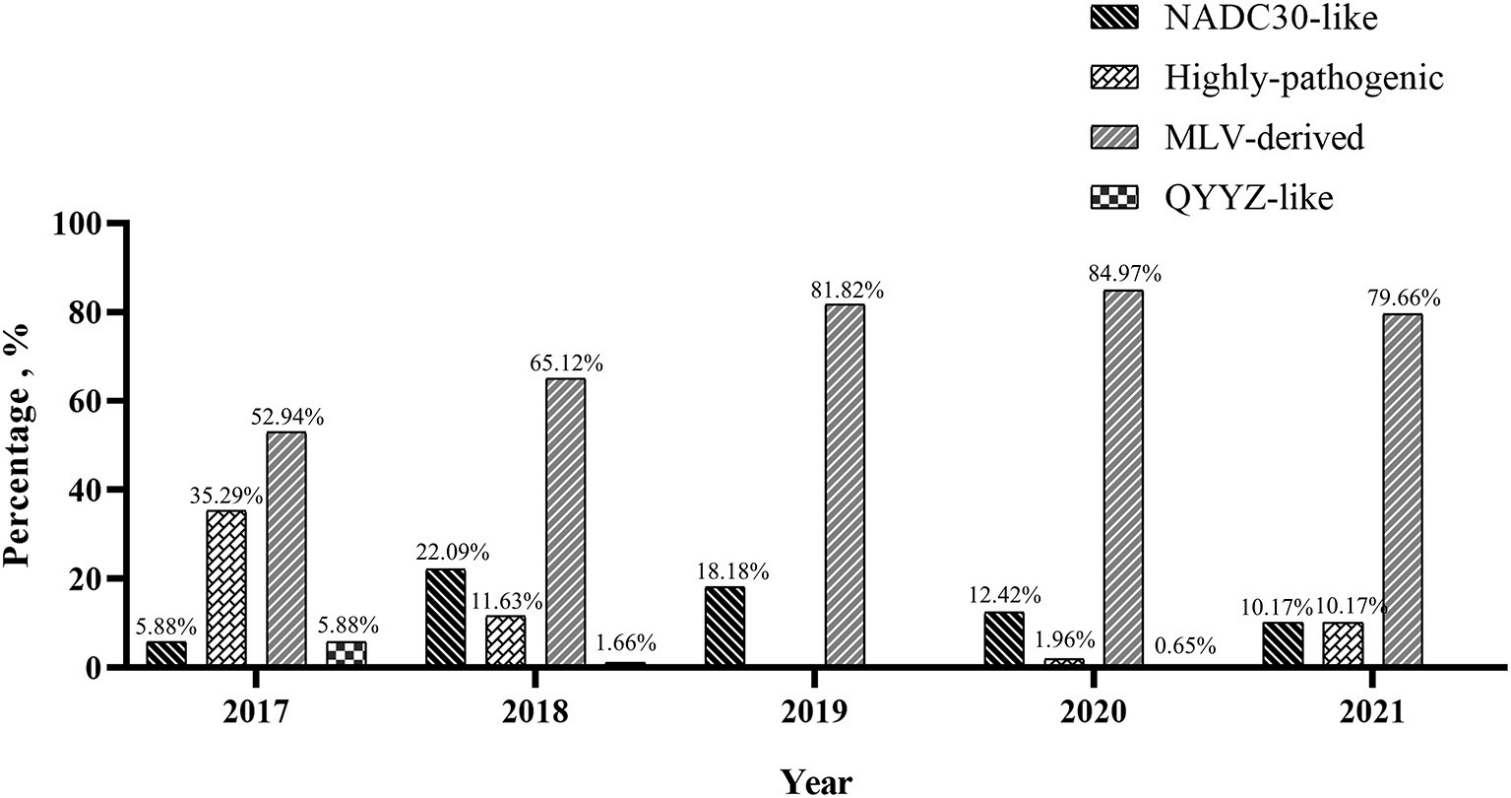
Annual detection rate of highly-pathogenic PRRSVs, MLV-derived PRRSVs, NADC30-like PRRSVs and QYYZ-like PRRSVs in South China between 2017 and 2021.

To understand the PRRSV type circulating in South China, we amplified and sequenced the ORF5 genes from 479 PRRSV positive samples and performed a phylogenetic analysis. The results revealed that all these 479 PRRSVs were PRRSV2 (Figure 2). Within the PRRSV2 group, ~85.39% (409/479) of the strains were highly-pathogenic PRRSVs which belonged to lineage 8, while 13.78% (66/479) of the strains were NADC30-like strains which belonged to lineage 1, and 0.84% (4/479) of the strains were QYYZ-like strains which belonged to lineage 3 (Figure 2). Among the 409 highly-pathogenic PRRSVs, 93.15% (381/409) of the strains were found to have a close relationship with the PRRSV modified vaccine-derived (MLV-derived) strains which are represented by TJbd14-1 (Accession no. KP742986) (30) and XJ17-4 (Accession no. MK759853) (31), with a mean nucleotide sequence identity of 92.5%. The annual detection rates of MLV-derived strains between 2017 and 2021 were 52.94% (9/17), 65.12% (56/86), 81.82% (9/11), 84.97% (260/306), and 79.66% (47/59), respectively, showing an increasing trend (Figure 3). However, the annual detection rates of NADC30-like strains between 2017 and 2021 were 5.88% (1/15), 22.09% (19/86), 18.18% (2/11), 12.42% (38/306), and 10.17% (6/59), respectively, showing a decreasing trend (Figure 3).

### Genomic Characteristics of the Eleven PRRSV Isolates

Eleven PRRSV strains were successfully isolated using PAM cells, one isolate (designated GD7666) was recovered from sample collected from Guangdong, the other ten isolates (designated GX505, GX1858, GX3251, GX4934, GX4853, GX5416, GX7111, GX7668, GX11045, and GX11373) was recovered from samples from Guangxi. Sequence comparison showed that the whole genome sequences of these eleven PRRSV isolates shared 81.1 to 87.5% nucleotide identity to that of the PRRSV2 representative strain VR2332, but only shared 57.5 to 58.7% nucleotide identity to that of the PRRSV1 representative strain Lelystad virus, suggesting that all the eleven isolates were PRRSV2 strains (Table 5). The genome sequences of six isolates (GX3251, GX4934, GX5416, GX7111, GX7668, and GD7666) had the highest nucleotide identities (99%, 99.2%, 94.3%, 98.8%, 98.8%, and 99.2%, respectively) with that of TJbd14-1 which was evolved from vaccine strain TJM-F92 (Accession no. MN508255). In addition, the genome sequences of two isolates (GX11045 and GX11373) showed the highest nucleotide identities (92.1 and 96.6%, respectively) with highly-pathogenic strain TJ. Notably, the genome sequences of the other three isolates (GX505, GX1858, and GX4852) had the highest nucleotide identities (89%, 88.7%, and 88.7%, respectively) with NADC30 (Table 5).

**Table 5:** Genomic nucleotide identity between the twelve isolates and PRRSV reference strains.

**Group**		**Virus** **(Genbank Accession No.)**	**Pairwise % genomic identity (nt)**										
			**GX11045**	**GX11373**	**GX1858**	**GX3251**	**GX4852**	**GX4934**	**GX505**	**GX5416**	**GX7111**	**GD7666**	**GX7668**
PRRSV1		LV (M96262)	57.6	57.5	58.5	58.4	58.7	58.4	58.7	58.1	58.2	58.3	58.4
PRRSV 2	Lineage 1	NADC30 (JN654459)	78.9	80	88.7	81.5	88.7	81.6	89	85.1	81.4	81.5	81.6
		SDQZ-1609 (MH651746)	78.9	79.8	88.3	81.3	88.3	81.4	88.6	84.7	81.2	81.2	81.4
		LNCH-1604 (MH651741)	78.8	79.7	88.5	81.3	88.5	81.4	88.8	84.6	81.1	81.2	81.4
		JL580 (KR706343)	81.2	83.3	88	84.9	88	85.1	88.3	88.2	84.8	84.9	85.1
		NADC34 (MF326985)	78.7	79.4	84.1	80.9	84.1	81	84.4	83	80.8	80.9	81
		NAV-Anheal-1 (MH370474)	78.3	79	83.5	80.5	83.6	80.6	83.9	82.6	80.4	80.4	80.6
	Lineage 3	QYYZ (JQ308798)	85.3	85.7	78.9	84.2	78.9	84.3	79.3	81.8	84	84.1	84.3
		GM2 (JN662424)	84.8	85.3	78.9	83.8	78.8	83.9	79.2	81.5	83.6	83.7	83.9
	Lineage 5	VR-2332 (PRU87392)	85.9	87.5	81.1	86.4	81.2	86.6	81.4	84.7	86.3	86.3	86.6
		RespPRRS_MLV (AP066183)	86.1	87.7	81.4	86.7	81.5	86.8	81.7	84.9	86.5	86.5	86.8
	Lineage 8	CH-1a (AY032626)	89.2	92.4	82.6	91.7	82.6	91.9	82.9	89	91.6	91.7	91.9
		CH-1R (EU807840)	89.1	92.4	82.6	91.7	82.6	91.8	82.9	88.9	91.5	91.6	91.8
		HuN (EF517962)	91.7	96.2	83.6	95.9	83.7	96.1	83.9	92.5	95.7	95.7	96.1
		TJ (EU860248)	92.1	96.6	84	96.2	84	96.4	84.3	92.9	96	96.1	96.4
		JXA1 (EF112445)	91.3	95.6	83.5	95.3	83.5	95.5	83.8	92	95.1	95.1	95.5
		XJ17-5 (MK75853)	89.5	94.4	84.9	98.8	84.9	99	85.2	94.1	98.6	98.6	99
		TJbd14-1 (KP742986)	89.6	94.6	84.9	99	84.9	99.2	85.2	94.3	98.8	98.8	99.2

### Nsp2 Sequence Analysis of the Eleven PRRSV Isolates

**Figure 4:**
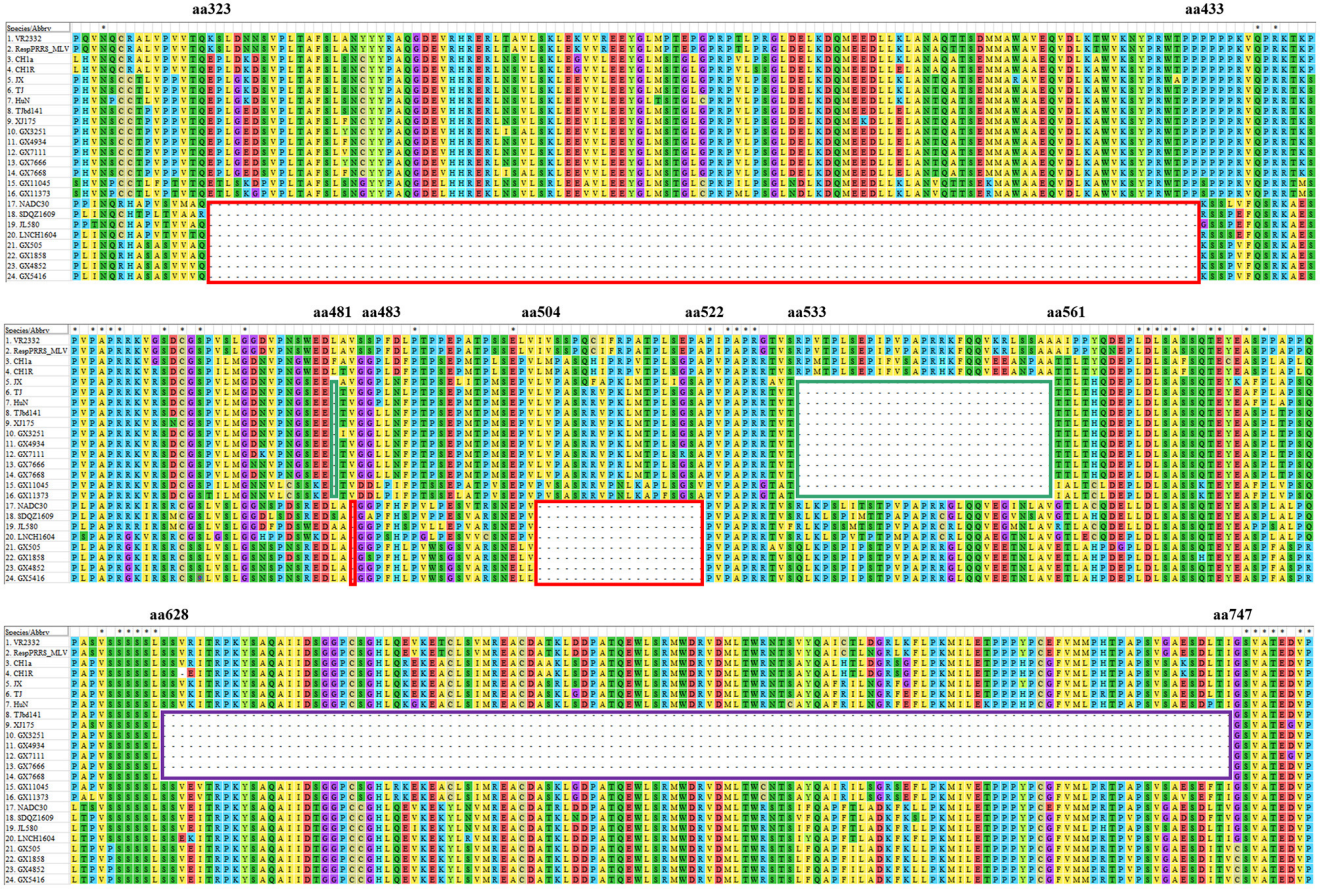
Alignment of the Nsp2 amino acid sequences of the eleven PRRSV isolates. The 131-amino-acid discontinuous deletion of NADC30-like PRRSVs is shown in red box. The 30-amino-acid discontinuous deletion of highly-pathogenic PRRSVs is shown in green box. The additional 120 amino-acid deletion of MLV-derived PRRSVs is shown in purple box.

Nsp2 region is one of the most variable regions in the genome of PRRSV. The nucleotide sequence identities of the Nsp2 genes among the eleven isolates ranged from 60.4 to 99.9%, and the amino acid similarities of the Nsp2 proteins ranged from 52.7 to 99.8%. Compared to the Nsp2 of VR2332, the Nsp2 proteins of four isolates (GX505, GX1858, GX4852, and GX5416) had a deletion pattern that was identical to those of the NADC30-like strains, all of them had a deletion of 131 amino acids at aa323-433, aa483, and aa504-522 (111 + 1+19) (Figure 4). However, the Nsp2 proteins of seven isolates (GX3251, GX4934, GX7111, GX7668, GX11045, GX11373, and GD7666) had an amino acid deletion pattern similar to that of the Chinese high-pathogenic strains, all of them possessed a deletion of 30 amino acids at aa481, and aa533-561 (1 + 29) compared to that of VR2332 (Figure 4). Particularly, five isolates (GX3251, GX4934, GX7111, GX7668, and GD7666) had a deletion of additional 120 amino acids at aa628-747 compared to the Nsp2 protein of VR2332, this amino acid deletion pattern was similar to that of the MLV-derived strains TJbd14-1 and XJ17-5 (Figure 4).

### ORF5 Sequence Analysis of the Eleven PRRSV Isolates

**Figure 5:**
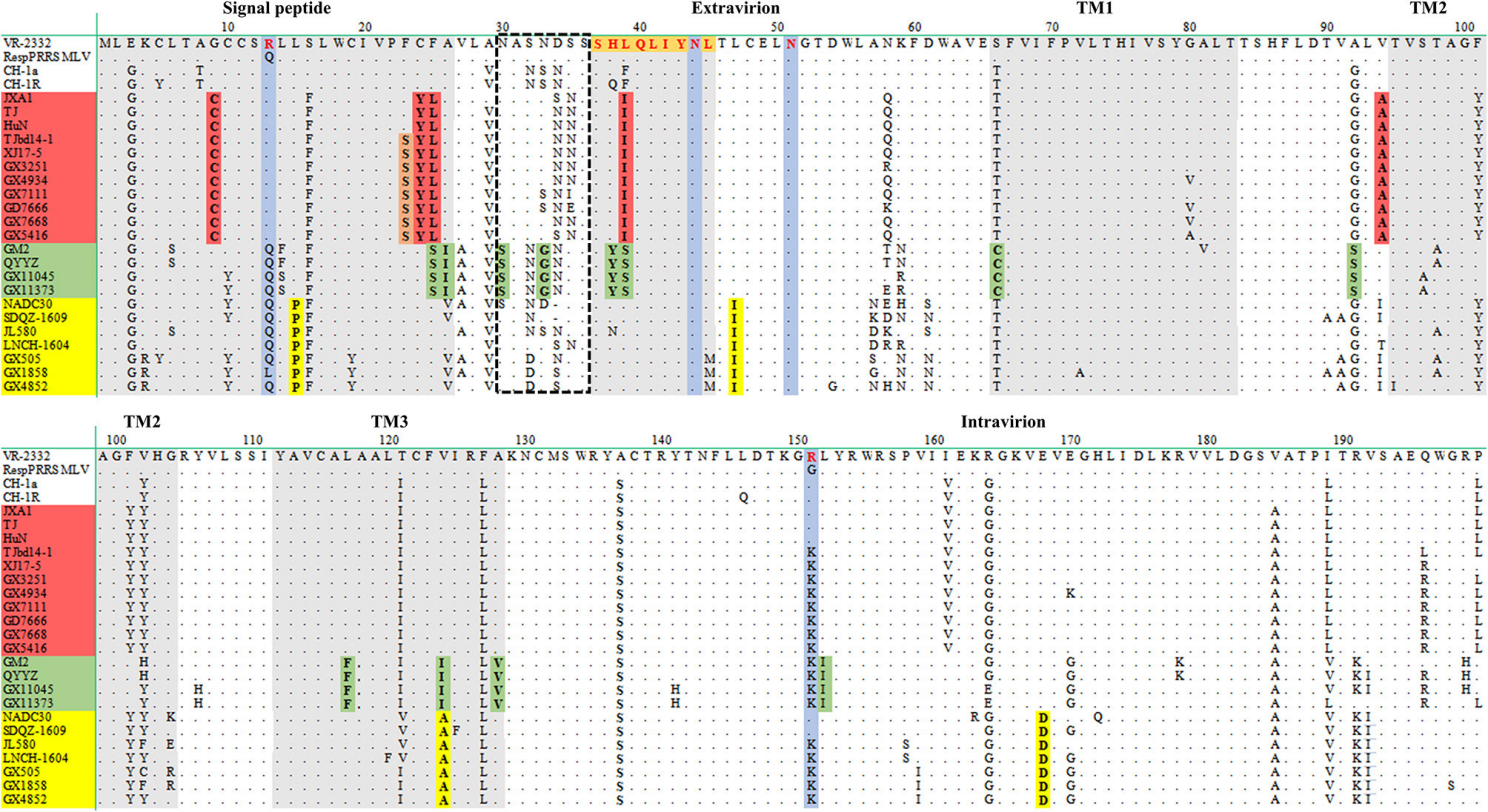
Alignment of the GP5 amino acid sequences of the eleven PRRSV isolates. The signal peptide, PNE and three transmembrane (TM) domains are shown in gray. Identical mutations of highly-pathogenic strains, QYYZ-like strains, and NADC30-like strains are shown in red, green and yellow, respectively. Variable region of NGSs is shown in dashed black box.

Sequence comparison of ORF5 genes showed that the nucleotide sequence identities of the ORF5 genes among the eleven isolates ranged from 81.1 to 99.8%, and the amino acid similarities of the GP5 proteins ranged from 77.6 to 100%. As shown in Figure 5, the most variable regions of GP5 protein were located in the extravirion region and signal peptide. The potential N-glycosylation sites (NGSs) at N44 and N51 were conserved in all isolates, but the potential NGSs located upstream of N44 were relatively variable. Compared to the GP5 protein of VR2332, three isolates (GX3251, GX4934, and GX7668) had mutations at sites 34 (D → N) and 35 (S → N), two isolates (GX7111 and GD7666) had mutations at sites 33 (N → S) and 34 (D → N), GX5416 had mutation at site 35 (S → N), two isolates (GX11045 and GX11373) had mutations at sites 30 (N → S), 32 (S → N), 33 (N → G) and 34 (D → N). However, the other three isolates (GX505, GX1858 and GX4852) showed no amino acid substitution of NGSs in this region.

Extensive mutations were observed in other areas of GP5 proteins. Compared to the GP5 protein of VR2332, all the isolates had a mutation at site 151 (R → K). Four isolates (GX11045, GX11373, GX505, and GX4852) had a mutation at site 13 (R → Q), and isolate GX1858 had a mutation at site 13 (R → L), while the other six isolates (GX3251, GX4934, GX5416, GX7111, GX7668, and GD7666) had a conserved R13 (Figure 5). Compared to VR2332, six isolates (GX3251, GX4934, GX5416, GX7111, GX7668, and GD7666) had amino acid changes in the signal peptide at sites 9 (G → C), 24 (C → Y), and 25 (F → L), the primary neutralizing epitope (PNE) of the extravirion region at site 39 (L → I), and the transmembrane (TM) region at site 94 (V → A) (Figure 5). Notably, this amino acid mutation pattern also occurred in the GP5 proteins of the Chinese highly-pathogenic strains. However, two isolates (GX11045 and GX11373) had amino acid changes in the signal peptide at sites 25 (F → S) and 26 (A → I), the extravirion region at sites 30 (N → S), 33 (N → G), 38 (H → Y), and 39 (L → S), the TM region at sites 66 (S → C), 92 (A → S), 117 (L → F), 124 (V → I), and 128 (A → V), and the intravirion region at site 152 (L → I) compared to the GP5 protein of VR2332. This amino acid mutation pattern also occurred in the GP5 proteins of GM2 and QYYZ (Figure 5). Finally, the other three isolates (GX505, GX1858, and GX4852) had amino acid changes in the signal peptide at site 15 (L → P), the TM region at sites 47 (L → I) and 124 (V → A), and the intravirion region at site 168 (E → D) compared to the GP5 protein of VR2332, and this amino acid mutation pattern also occurred in the GP5 proteins of the NADC30-like strains (Figure 5).

### Phylogenetic Analysis of the Eleven PRRSV Isolates

**Figure 6:**
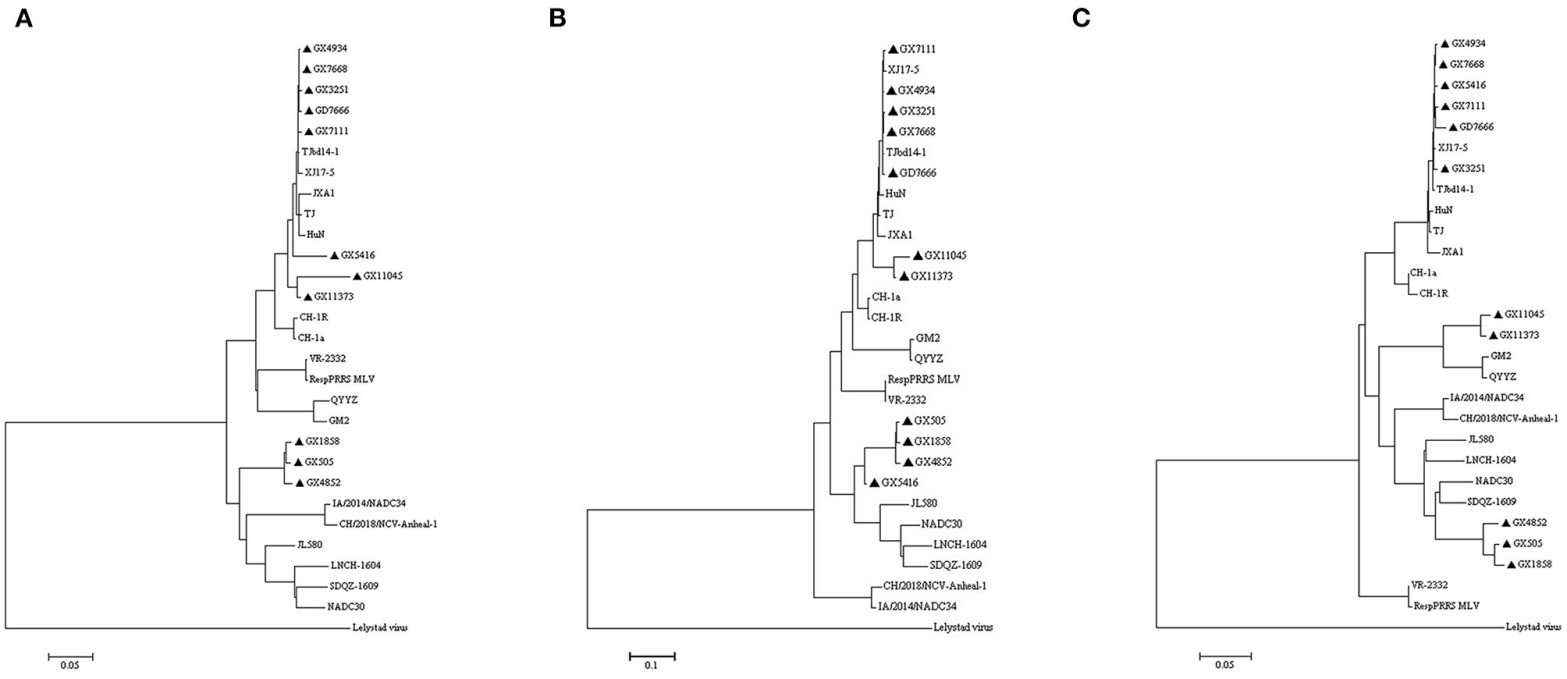
Phylogenetic trees based on the complete genome, nsp2, and ORF5 of PRRSV. **(A)** Complete genome nucleotide; **(B)** Nsp2 nucleotide; **(C)** ORF5 nucleotide. The isolates recovered in this study are indicated by black triangles.

To understand the phylogenetic characteristics of all the eleven PRRSV isolates, we generated three phylogenetic trees based on complete genome sequences, Nsp2 nucleotide sequences, and ORF5 nucleotide sequences, respectively (Figures 6A–C). According to the phylogenetic tree generated based on the whole genome sequences (Figure 6A), eight isolates (GX3251, GX4934, GX5416, GX7111, GX7668, GX11045, GX11373, and GD7666) clustered in lineage 8 with highly-pathogenic PRRSVs TJ, JXA1, and HuN, three isolates (GX1858, GX505, and GX4852) clustered into lineage 1 which was represented by NADC30 and NADC34. Interestingly, GX5416 clustered into lineage 1 together with NADC30 in the phylogenetic tree generated based on the Nsp2 gene (Figure 6B), while GX11045 and GX11373 clustered into lineage 3 together with QYYZ and GM2 based on the ORF5 gene (Figure 6C), indicating that these strains might undergo recombination events.

### Recombination Analysis of the Eleven PRRSV Isolates

**Figure 7:**
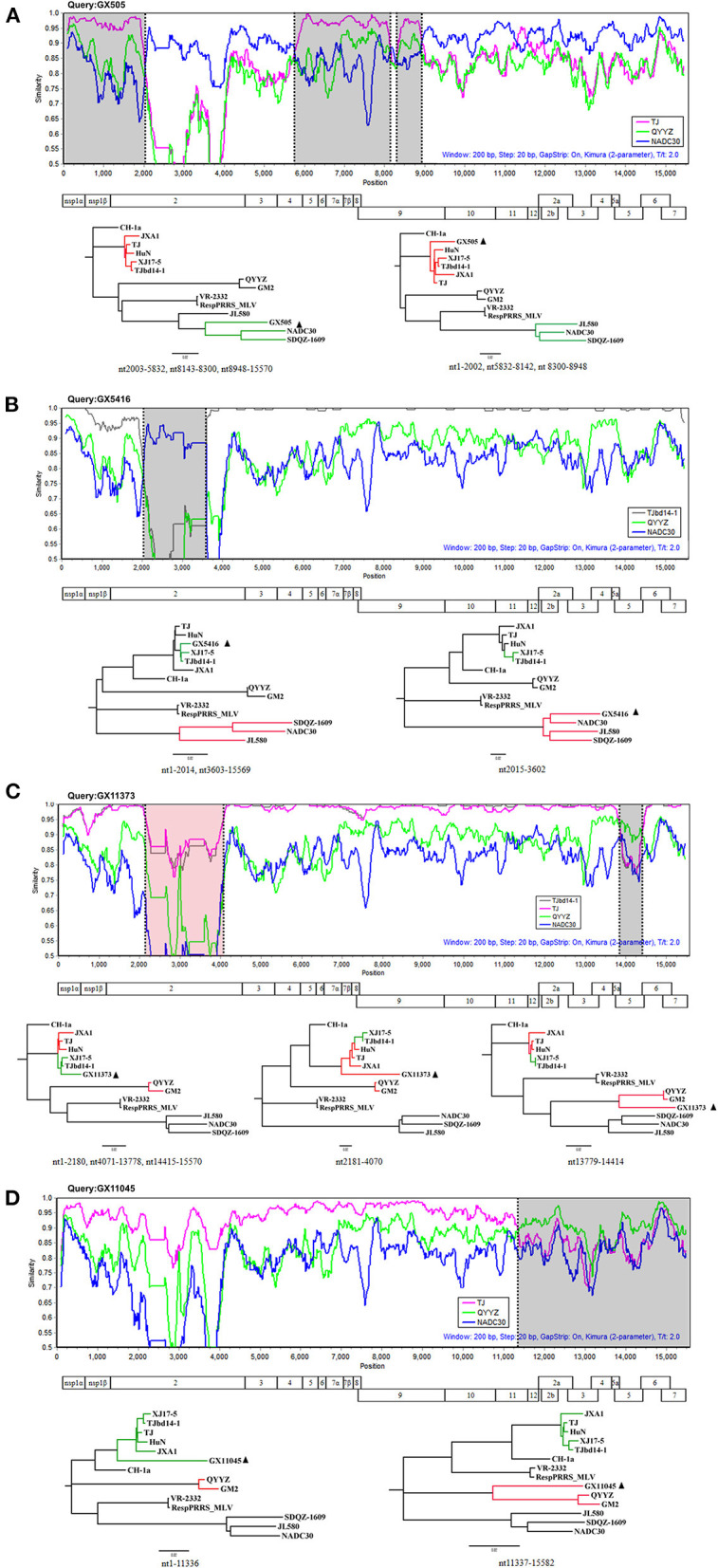
Recombination analysis of strains GX505 **(A)**, GX5416 **(B)**, GX11373 **(C)**, and GX11045 **(D)**. Genome scale similarity comparison of the isolates (query) with TJ (orange), TJbd14-1 (gray), NADC30 (blue), and QYYZ (green). Recombination breakpoints are shown as black dashed lines. The background color of the major parental regions is white, whereas that of the minor parental regions is gray or red. Below the similarity plot is a full genome structure of PRRSV, with reference to CH-1a, shows the position of the ten open reading frames and the fourteen Non-structural proteins. Phylogenetic trees based on different regions of the isolates are shown below the similarity plots. The major parental group is shown in green, the minor parental group is shown in red. The isolates recovered in this study are indicated by black triangles.

Recombination events were detected using RDP software according to the full-length genome sequences and the results were confirmed by SIMPLOT. The results revealed that the genomes of six PRRSV isolates (GX505, GX1858, GX4852, GX5416, GX11373, and GX11045) were identified with recombination events (Table 6). Among these six isolates, three isolates (GX505, GX1858, and GX4852) shared the same recombination pattern (Table 6). Take isolate GX505 for example, it possessed five recombination breakpoints located in Nsp2 (nt2002), Nsp4 (nt5832), and Nsp9 (nt8142, nt8300, and nt8948). These breakpoints separated the genome of GX505 into six regions. Of them, three regions were closely related to TJ (highly-pathogenic PRRSV), and the remaining three regions were similar to NADC30, suggesting that GX505 was recombined from NADC30-like strain and highly-pathogenic PRRSV (Figure 7A). In addition, the genome of GX5416 had two recombination breakpoints located in Nsp2 (nt2014 and nt3602) which divided the genome into three segments. The intermediate segment was similar to NADC30 and the remaining two segments were closely related to TJbd14-1 (MLV-derived strain), indicating that GX5416 was a recombinant strain between MLV-derived strain and NADC30-like strain (Figure 7B). The genome of GX11373 had four breakpoints located in Nsp2 (nt2180 and nt4070) and ORF5 (nt13778 and nt14414) which divided the genome into five segments. Segment nt13779-14414 clustered with QYYZ, and segments nt1-2180, nt4071-13778, and nt14415-15570 clustered with TJbd14-1 (MLV-derived strain), while another unknown minor parent strain provided the nt2181-4070 within Nsp2. The phylogenetic analysis showed that region nt2181-4070 of GX11373 was closely related to highly-pathogenic PRRSVs. Therefore, we speculated that GX11373 was a recombinant strain from MLV-derived strain, QYYZ-like strain, and highly-pathogenic strain (Figure 7C). The genome of GX11045 had one recombination breakpoint located in Nsp11 (nt11336). Phylogenetic trees showed that it was recombination product between highly-pathogenic PRRSV and QYYZ-like strain (Figure 7D).

**Table 6:** Information of recombination events of the isolated PRRSV.

**Isolates**	**Major** **(similarity)**	**Minor** **(similarity)**	**Breakpoints**		**Average** ***p*****-value of the detection methods**						
			**Beginning**	**Ending**	**RDP**	**GENECONV**	**BootScan**	**MaxChi**	**Chimera**	**SiScan**	**3Seq**
GX505	NADC30 (90.1%)	TJ (97.1%)	1	2,002	1.436 × 10^−79^	2.790 × 10^−74^	2.842 × 10^−59^	6.394 × 10^−35^	3.426 × 10^−20^	2.647 × 10^−39^	2.442 × 10^−14^
	NADC30 (91.4%)	TJ (97.2%)	5,832	8,142	3.885 × 10^−104^	2.374 × 10^−97^	1.072 × 10^−103^	3.287 × 10^−31^	4.924 × 10^−22^	2.552 × 10^−40^	5.372 × 10^−06^
	NADC30 (91.8%)	TJ (96.6%)	8,300	8,948	2.003 × 10^−31^	4.776 × 10^−22^	2.011 × 10^−24^	2.953 × 10^−10^	2.494 × 10^−11^	3.455 × 10^−11^	9.769 × 10^−14^
GX1858	NADC30 (90.1%)	TJ (96.9%)	1	2,002	2.489 × 10^−79^	5.464 × 10^−73^	9.507 × 10^−73^	7.045 × 10^−34^	2.788 × 10^−37^	4.751 × 10^−39^	1.730 × 10^−08^
	NADC30 (91.4%)	TJ (97.2%)	5,834	8,142	5.469 × 10^−105^	1.586 × 10^−97^	2.000 × 10^−108^	1.658 × 10^−31^	6.340 × 10^−22^	3.978 × 10^−41^	9.769 × 10^−14^
	NADC30 (91.8%)	TJ (96.2%)	8,302	8,910	1.650 × 10^−30^	1.376 × 10^−19^	1.383 × 10^−28^	2.212 × 10^−10^	5.729 × 10^−11^	2.640 × 10^−10^	9.769 × 10^−14^
GX4852	NADC30 (91.2%)	TJ (96.2%)	1	2,002	1.090 × 10^−93^	3.207 × 10^−77^	4.385 × 10^−75^	3.888 × 10^−32^	2.100 × 10^−18^	7.884 × 10^−37^	9.769 × 10^−14^
	NADC30 (89.7%)	TJ (97%)	5,832	8,142	8.677 × 10^−74^	4.490 × 10^−71^	2.038 × 10^−71^	5.150 × 10^−31^	4.343 × 10^−14^	4.934 × 10^−40^	7.327 × 10^−14^
	NADC30 (91.6%)	TJ (96%)	8,300	8,948	4.643 × 10^−29^	2.138 × 10^−18^	NS	9.806 × 10^−10^	2.233 × 10^−11^	1.202 × 10^−10^	5.329 × 10^−14^
GX5416	TJbd14-1 (98.8%)	NADC30 (90.7%)	2,014	3,602	1.200 × 10^−161^	5.855 × 10^−111^	6.731 × 10^−154^	1.340 × 10^−35^	3.026 × 10^−38^	9.296 × 10^−32^	2.424 × 10^−13^
GX11045	QYYZ (89.1%)	TJ (94.6%)	1	11,336	1.504 × 10^−15^	8.526 × 10^−20^	6.705 × 10^−16^	1.210 × 10^−09^	4.120 × 10^−20^	2.891 × 10^−63^	3.694 × 10^−36^
GX11373	TJbd14-1 (99.2%)	Unknown	2,180	4,070	1.471 × 10^−15^	1.143 × 10^−11^	2.184 × 10^−12^	5.925 × 10^−19^	2.376 × 10^−19^	1.516 × 10^−18^	4.041 × 10^−14^
	TJbd14-1 (98.1%)	QYYZ (92.3%)	13,778	14,414	1.701 × 10^−49^	1.958 × 10^−26^	6.893 × 10^−50^	1.487 × 10^−17^	7.588 × 10^−15^	1.052 × 10^−08^	2.020 × 10^−13^

## Discussion

Since its first emergence in China in 1996, PRRS has been widely spreading and continuing to evolve rapidly in China, causing significant economic losses in the world's largest pig-raising and pork-consuming country (18, 32, 33). As reported in previous studies, the positive rate of PRRSV in South China varied between years. In Guangxi, the detection rates were 35.12% between 2013 and 2014 (34), 57.14% in 2016 (26), 12.62% between 2017 and 2018 (12), and 29.54% between 2020 and 2021 (35). In this study, the detection rate ranged from 4.92 to 24.63% in Guangxi between 2017 and 2021. In Guangdong, the detection rates were 16.60% between 2007 and 2011 (36), 32.54% between 2010 and 2013 (37), and 40% between 2017 and 2018 (12). In our study, the detection rate ranged from 24.90 to 52.94% in Guangdong between 2017 and 2021. However, we found that the overall detection rates in South China showed a yearly increasing trend from 2017 to 2021, as detection rate in 2019 was less representative due to limited sample size, and maintained at a high level every month in 2020 and 2021. This might be associated with the prevalence of African swine fever (ASF) in this region. ASF first emerged in South China by end of 2018, causing a devastating impact on the swine industry in that region in 2019 (38, 39). Those swine-raising companies started to resume production in 2020 and 2021. Lots of gilts were transported, inducted, and reared together from farms with different statuses of PRRS, which led to a yearly rising detection rate of PRRSV. Outbreaks of ASF also resulted in a sharp decrease in sample size in 2019. On the one hand, the emphasis of disease control in pig farms has been shifted from disease diagnosis to biosecurity against a background of ASF. On the other hand, pig farms saw autopsy and sampling of diseased pigs as risks of spreading pathogens.

It has been reported that PRRSV strains circulating in China were mainly PRRSV2 (12, 40). Consistently, all of the 479 ORF5 sequences detected from 2017 to 2021 were PRRSV2. Particularly, our results demonstrated that the highly-pathogenic strains remained the predominant PRRSVs in South China, which are in agreement with those of the previous publications (26, 36, 37, 41, 42). It should be noted that NADC30-like strains were the other main type circulating in South China from 2017 to 2021 and exhibited a yearly decreasing detection rate which was reported for the first time to the best of my knowledge.

We also found that 93.15% of the highly-pathogenic PRRSVs were PRRSV MLV-derived strains with a yearly increasing detection rate from 2017 to 2021. A retrospective survey found that highly-pathogenic PRRSV MLV vaccines have been widely used in South China. These findings suggested that the prevalence of highly-pathogenic PRRSV in South China might be largely related to the use of PRRSV MLV vaccines. Several commercial MLV vaccines have been inducted to and widely used in Chinese swine herds (43–45), the virulence reversion of vaccine strains has been reported continuously ever since (29, 46, 47), and lots of MLV-derived strains have been isolated from Chinese swine herds (31, 48–50). Of the eleven PRRSV isolates in our study, seven isolates (GX3251, GX4934, GX5416, GX7111, GX7668, GX11373, and GD7666) were found to be MLV-derived. It should be noted that it is still controversial whether PRRSV MLV vaccines should be used. According to many reports, the efficiency of PRRSV MLV vaccines was not significant and they provided only limited protection in the field (51–55), as PRRS still outbroke in many MLV-vaccinated farms (44, 55–57). Therefore, we strongly recommend that priority should be given to bio-security and management measures rather than vaccines when controlling PRRS.

Mutations play important roles in PRRSV evolution. Amino acid deletion patterns in Nsp2 proteins compared to that of VR2332 were recognized as genetic markers of certain genotypes of PRRSVs (12). In our study, seven isolates (GX3251, GX4934, GX7111, GX7668, GX11045, GX11373, and GD7666) had an amino acid deletion pattern of discontinuous deletion of 30aa (1 + 29) that was identical in highly-pathogenic PRRSVs (26, 58, 59), and four isolates (GX 505, GX1858, GX4852, and GX5416) had a deletion pattern of discontinuous deletion of 131aa (111 + 1+19) which was identical to NADC30-like strains (23, 24). Besides, five isolates (GX3251, GX4934, GX7111, GX7668, and GD7666) had an additional deletion of continuous 120aa which was recognized as a molecular marker of TJM-F92 vaccine strain (48, 60). It has been reported that deletions in Nsp2 might eliminate dispensable genomic regions to make the genome more compact which might benefit the survival of the strains in the field (48, 61).

Multiple amino acid mutations have also been found in the GP5 proteins of highly-pathogenic strains, GM2-like strains, and NADC30-like strains compared to those of classic PRRSV strains represented by VR2332 (12, 14, 60). Mutations commonly occurred in the signal peptide coding region (aa1-26), primary neutralizing epitope (PNE) in the extravirion region (aa37-45), and transmembrane (TM) regions (aa66-83, aa95-104, and aa112-128) of GP5 protein (12, 36, 62). H38, I42, Y43, and N44 residues are recognized as main antigen recognition sites of GP5 protein, while L39, Q40, and L41 residues are recognized as antibody binding sites (7, 60, 63–65). The PNE sequence of VR2332 is 37-SHLQLIYNL-45. In our study, six isolates (GX3251, GX4834, GX5416, GX7111, GX7668, and GD7666) had a mutation at site 39 (L → I) which was also found in highly-pathogenic PRRSVs TJ, JXA1, HuN. However, two isolates (GX11045 and GX11373) had mutations at sites 38 (H → Y) and 39 (L → S) which were consistent with QYYZ and GM2, and three isolates (GX505, GX4852, and GX1858) had a mutation at site 45 (L → M) which was also found in NADC30-like strains. These amino acid substitutions in the PNE region might lead to the failure of receptor recognition and thus result in the failure of vaccines. GP5 of PRRSV2 contains 3 to 5 potential N-glycosylation (NGS) sites, of which N44 and N51 are strongly conserved, while NGSs located upstream of N44 are relatively variable and important for the virus to escape antibody neutralization (26, 41, 66–68). All the eleven isolates in our study had conserved N44 and N51. In addition, three isolates (GX3251, GX4934, and GX7668) had another five NGSs, isolate GX5416 had another four NGSs, three isolates (GX505, GX7111, and GD7666) had another three NGSs, and four isolates (GX1858, GX4852, GX11045, and GX11373) had another two NGSs located upstream of N44. These findings suggested that PRRSVs have undergone rapid evolution in the field in South China. It has been reported that the high number of potential NGSs might contribute to minimize the immunogenicity of the nearby neutralizing epitope, which might enhance the virus to escape the recognition of immune system and avoid neutralization effects induced by PRRSV MLV vaccines (69). Our findings are also consistent with previous research results showing that the number of potential NGSs has increased in Chinese isolates over time (70). R13 and R151 residues of GP5 were reported to be associated with PRRSV virulence, and amino acid substitutions at these sites might lead to attenuation (14, 60). In our study, all the isolates had a mutation at site 151 (R → K). In addition, four isolates (GX11045, GX11373, GX505, and GX4852) had a mutation at site 13 (R → Q), and R13 of isolate GX1858 mutated into 13L, while the other six isolates exhibited a conserved R13. Further research should be conducted to detect whether these mutations lead to virulence change.

Recombination events commonly occurred among different PRRSV isolates and were considered as one of the most principal mechanisms in PRRSV diversification and evolution (17, 30). In agreement with these findings, six of the eleven isolates recovered in this study showed evidence of recombination events in the genomes. Compared to the other PRRSV lineages, NADC30-like strains show more recombination possibilities (19, 71). Recombination events between NADC30-like strains and PRRSVs from other lineages have been reported (20, 23, 62, 72–76). Among the six recombinant isolates in our study, three isolates (GX505, GX1858, GX4852) were recombination products between NADC30-like strains and highly-pathogenic PRRSVs, while isolate GX5416 was recombined from NADC30-like strain and MLV-derived strain. The other two isolates (GX11045 and GX11373) were all related to QYYZ-like strains which circulated in South China since its emergence in 2010 (21). These findings suggested that recombination is still happening at high frequency among different PRRSVs circulating in South China and has led to the increase of genetic diversity and complexity of PPRSV in this region. It is worth noting that two isolates (GX5416 and GX11373) in our study were found to be recombinants between MLV-derived strains and PRRSVs from other lineages. Since recombinations between PRRSV MLV-derived strains and field strains have been largely reported in China (21, 72, 75–80), more attention should be drawn to the restrained application of PRRSV MLV vaccines.

Interestingly, some isolates were recovered from different geographical regions but shared high homology and similar genetic characteristics. For example, GX505, GX1858, and GX4852 were isolated from three different sow farms with hundred kilometers away from each other, but they exhibited high nucleotide identities of more than 98% and the same recombination pattern. We found that these three sow farms shared the same feed plant. We speculated that the virus induction may be attributed to feed transportation among the three farms. These findings suggested that long-range transmission of PRRSV may also happen which urges the farm to pay more attention to bio-security when controlling diseases.

In conclusion, we investigated the epidemiological status of PRRSV in South China between 2017 and 2021. We found that highly-pathogenic PRRSV remained the predominant PRRSV type in the field. Our findings also suggested that highly-pathogenic PRRSV strains in the field might be mainly MLV-derived, and the use of PRRSV MLV vaccines in the field should receive more attention. In addition, a number of mutations has been found within Nsp2 and GP5 proteins. We also found that recombination among different PRRSV strains was common in the field, which may exert great pressure on PRRS control.

## Data Availability Statement

The raw data supporting the conclusions of this article will be made available by the authors, without undue reservation.

## Ethics Statement

All samples used in this study were sent and provided by the farm owner. This study does not involve in human or animal use.

## Author Contributions

KF and PQ: conceived and designed research. KF, SL, and PQ: conducted experiments and analyzed data. KF: wrote the manuscript. XL, HC, and PQ revised the manuscript. All authors have read and approved the manuscript.

## Funding

This work was supported by the National Program on Key Research Project of China (2018YFD0500801 and 2018YFD0500204).

## Conflict of Interest

The authors declare that the research was conducted in the absence of any commercial or financial relationships that could be construed as a potential conflict of interest.

## Publisher's Note

All claims expressed in this article are solely those of the authors and do not necessarily represent those of their affiliated organizations, or those of the publisher, the editors and the reviewers. Any product that may be evaluated in this article, or claim that may be made by its manufacturer, is not guaranteed or endorsed by the publisher.
